# Trigonella foenum-graecum Methanolic Extract on Isolated Smooth Muscles and Acetylcholinesterase Enzyme: An In Vitro and Mechanistic In Silico Investigation

**DOI:** 10.1155/2022/4849464

**Published:** 2022-04-05

**Authors:** Muhammad Nabeel Ghayur, Mohnad Abdalla, Asaad Khalid, Saeed Ahmad, Anwarul Hassan Gilani

**Affiliations:** ^1^Kentucky College of Osteopathic Medicine, University of Pikeville, Pikeville, 41501 Kentucky, USA; ^2^Department of Biological and Biomedical Sciences, Aga Khan University, Karachi, 74800 Sind, Pakistan; ^3^Key Laboratory of Chemical Biology (Ministry of Education), Department of Pharmaceutics, School of Pharmaceutical Sciences, Cheeloo College of Medicine, Shandong University, 44 Cultural West Road, Shandong Province 250012, China; ^4^Substance Abuse & Toxicology Research Center, Jazan University, Jazan 45142, Saudi Arabia; ^5^Medicinal and Aromatic Plants and Traditional Medicine Research Institute, National Center for Research, Khartoum 11111, Sudan; ^6^Dept of Public Health and Nutrition, The University of Haripur, Haripur 22620, Khyber Pakhtunkhwa, Pakistan

## Abstract

**Results:**

When tested on the baseline of isolated tissues, Tfg.Cr was devoid of any activity (stimulant or relaxant) till 10 mg/ml. This is an interesting finding, keeping in mind that the fenugreek seeds are used to alleviate constipation and diarrhoea. When Tfg.Cr was tried for any potential AChE inhibitory activity, it did show an inhibitory effect in increasing concentrations (47-380 *μ*g/ml). This inhibitory effect was comparable to the effect produced by a standard AChE inhibitor physostigmine. One of the known fenugreek constituents, diosgenin, was also tested, and it also showed an AChE inhibitory effect in a concentration-dependent manner (11-190 *μ*g/ml). Interaction between diosgenin and AChE was further investigated by molecular docking and molecular dynamics simulations for 100 ns, which showed that diosgenin interacted with the active-site gorge of AChE through hydrophobic, pi-pi stacking, and hydrogen bonds with various amino acids of the AChE enzyme.

**Conclusion:**

The results show that the fenugreek extract does not possess any GI stimulant or relaxant activity even though it is used traditionally in GI motility disorders. The extract and diosgenin could inhibit the AChE enzyme pointing towards their benefit to enhance the memory.

## 1. Introduction

Trigonella foenum-graecum Linn. (family: Fabaceae or Leguminosae), or “fenugreek” as commonly known in English, is a widely used medicinal plant. In Urdu or Hindi, it is called “methi.” An annual herb [1, 2], fenugreek is originally from southeast Europe (the Mediterranean region) and West Asia [3, 4], but today, it is cultivated in many areas of the globe, including South Asia [2], Northern Africa, and even in North America [3]. It is a known and often consumed condiment all over the world [3]. In terms of its traditional use, the whole plant, leaves, and seeds are employed for medicinal benefits [1, 2]. The active medicinal compounds and the most potent activity are concentrated in the oblong, yellow to yellowish brown seeds [3, 4].

For centuries, fenugreek has popularly been used by herbalists and traditional healers of China and South Asia in several medical conditions, specifically for gastrointestinal (GI) and neurological issues. The seeds are consumed as is or processed (boiled or roasted) for their benefit in dyspepsia, colic, flatulence, diarrhoea, dysentery, anorexia [1, 2, 4], gastritis, constipation [4], chronic cough, and bronchitis [2, 3]. It is also used as a stimulant and tonic of the central nervous system (CNS) [1] and is known to enhance memory [5]. There are also claims for its activity in the treatment of all kinds of skin infections and inflammatory conditions (leg ulcers, wounds, abscess, cellulitis, boils, and carbuncles), myalgia, arthritis, kidney, and liver problems [1–4] and as a galactagogue [1, 6]. Despite the popularity of fenugreek, most of these above-mentioned claims await verification and scientific proof [4]. Some of the studies done on fenugreek report that it has galactagogue [7, 8], anti-inflammatory [9, 10], antidiabetic [10], anticholesterolemic [11, 12], antihypertensive, kidney and liver protective [13], androgenic/anabolic [14], antibacterial, and anticancer [15] properties. All these medicinal uses and properties show how popular and beneficial this herb is in such a broad spectrum of disease conditions.

Many of the benefits of this plant are linked to the chemicals that have been isolated from it. Fenugreek is known to contain steroidal saponins like diosgenin, tigogenin, trigogenin, and fenugreekine; alkaloids like choline and trigonelline; amino acids like histidine, arginine, tryptophan, and lysine; and vitamins like nicotinic acid [1, 2, 4, 16]. The steroidal saponins are responsible for most of the medicinal benefits of fenugreek [3].

**Figure 1:**
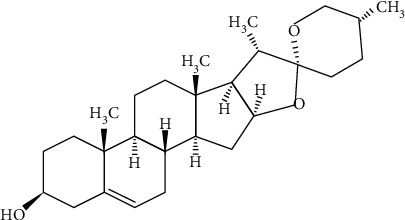
The chemical structure of fenugreek active constituent, a steroidal sapogenin, diosgenin (European Pharmacopoeia (EP) Reference Standard, structure taken from website of source of the chemical: https://www.sigmaaldrich.com).

As noted above, several experimental and clinical studies on fenugreek scientifically elucidate its traditional uses. But still, a lot more work needs to be done to discover the many hidden benefits of this herb. This was the idea behind undertaking this endeavour. A 70% aqueous methanolic crude extract was prepared. This extract was tested pharmacologically on gastrointestinal (GI) smooth muscle preparations from rabbits and rats to see if there is any smooth muscle tone modulatory activity in the GI system, a potential acetylcholine- (ACh-) like effect that would lead us to believe in its memory-enhancing effects too. A substance acting like ACh can potentially have both GI stimulant and memory-enhancing effects due to an action similar to ACh on the cholinergic receptors in the GI tract and CNS. The extract was also tested for activity against the acetylcholinesterase (AChE) enzyme in vitro. This is because fenugreek is used to enhance the memory. Although the extract did not exhibit any effect on GI smooth muscle preparations, it did show an inhibitory effect on the AChE enzyme. The AChE inhibitory activity was traced back to its known chemical ingredient, diosgenin (Figure 1). Interaction between diosgenin and AChE was further investigated by molecular docking and molecular dynamics simulations for 100 ns.

## 2. Methodology

### 2.1. Animals

Care was taken to avoid any suffering to the animals used in this study. Experiments were performed ethically in strict accordance with lab animal handling specifications of European Community guidelines, EEC Directive 86/609/EEC. Local rabbits (either sex, around 1 kg) and Sprague-Dawley rats (either sex, 170-200 g) were used in this project. These were kept in the animal quarter at the Aga Khan University. The air was pathogen-free, and the temperature was controlled at around 23°C. The rabbits and rats had free access to water, although the food was withheld a day before the experiments. Food given to the animals was made of the following: fiber, table salt, white flour, sweetener, Nutri-vet L, potassium metabisulfite, grease, seafood, and powdered milk.

### 2.2. Chemicals

Standard chemicals and reagents were obtained from Sigma Company, USA. These included acetylcholine (ACh), acetylthiocholine (ATCh), 5,5-dithiobis (2-nitro), benzoic acid (DTNB), diosgenin, electric eel AChE (type VI-S), histamine, nicotine, and physostigmine. The solutions and dilutions of these were made on the day of the experiment. To make Tyrode's physiological salt solution, chemicals were purchased from Sigma, USA, and Merck, Germany. Tyrode's was constituted as follows (mM): 2.68 KCl, 136.90 NaCl, 1.05 MgCl_2_, 11.90 NaHCO_3_, 0.42 NaH_2_PO_4_, 1.80 CaCl_2_, and 5.55 glucose.

### 2.3. Fenugreek Seeds and the Process of Extract Making

**Figure 2:**
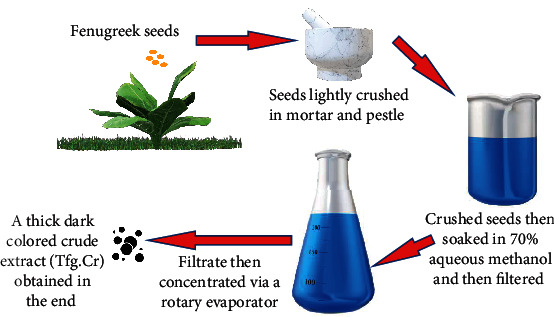
Schematic representation of the plant material (fenugreek seeds) and the crude extract preparation. All pictures in the scheme were taken from 3D models, Online Sources, Microsoft PowerPoint 2019.

Fenugreek seeds (around a kg) were acquired from a supplier in Karachi, Pakistan. This was botanically identified by Mr. S. Ahmad. A specimen was kept in the herbarium of Natural Products Research Unit, Aga Khan University, for identification and cataloguing (# TF-SE-05-04-59). For making the crude extract, methodology described previously [17] was used (Figure 2). Briefly, seeds were washed with water and then lightly mashed. The seeds were then kept dipped for 3 days in a couple of litres of 70% aqueous methanol at 23°C. After the 3 days, the seeds and methanol were passed through a fabric filter. Later, the plant material was immersed again in a new batch of aqueous methanol for 72 hours, twice. Finally, filtrates were filtered using Whatman-qualitative grade-1 filters. A rotary evaporator (Rotavapor, BUCHI Labortechnik AG, Switzerland) was used to obtain a crude extract, labelled as Tfg.Cr. We kept this extract stored at -4°C until use.

### 2.4. Experiments on Isolated Smooth Muscle Tissue Preparations

#### 2.4.1. Isolated Rabbit Jejunum

**Figure 3:**
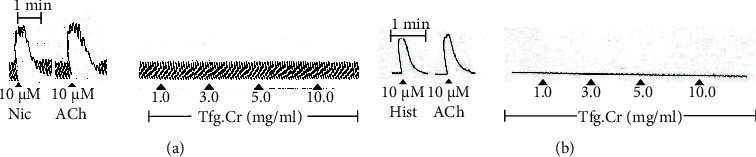
Tracings showing the effect of fenugreek crude extract (Tfg.Cr) on isolated intestinal tissue preparations. (a) Activity of Tfg.Cr on resting spontaneous contractions of rabbit jejunum tissues in comparison to standard drugs like acetylcholine (ACh) and nicotine (Nic). (b) Activity of Tfg.Cr on resting baseline of isolated rat ileum tissues in comparison to standard drugs like acetylcholine (ACh) and histamine (Hist).

We have described and worked with isolated intestinal smooth muscles before. Detailed descriptions can be found in our previous communication [17]. Portions of the jejunum (around 2 cm long) were obtained from rabbits. These were hung in tissue baths with a thread. The tissue baths were filled with the physiologic salt solution, aerated with a mixture of 95% O_2_ and 5% CO_2_ at physiologic temperature. All changes in contractility were noted isotonically using Harvard equipment (oscillographs and force transducers). The tissues were left to stabilize for half an hour. The advantage of using rabbit jejunum is its ability to contract spontaneously (Figure 3(a)). This permits to test the spasmolytic and spasmogenic potential of a drug being investigated. The spasmolytic effect is calculated as the % change in contractility of the tissue, while the spasmogenic effect is quantified while comparing with the action of a standard spasmogenic agent like ACh 10 *μ*M and nicotine 10 *μ*M (Figure 3(a)).

#### 2.4.2. Isolated Rat Ileum

We have previously described the use of isolated smooth muscle preparations [17]. Small intestinal sections of the ileum (about 2 cm long) were obtained from rats. These pieces of the tissue were hung in baths as described above. Changes in contractility of the tissue were captured isotonically using Harvard equipment. The tension kept on the tissue was 1 g. Unlike rabbit jejunum, rat ileum does not exhibit baseline contractions; instead, it has a flat baseline (Figure 3(b)). The tissue was left to normalize for half an hour, following which repeated contractions were obtained from standard drugs like ACh 10 *μ*M and histamine 10 *μ*M (Figure 3(b)). Drugs were permitted to stay in contact with tissue for around 20 s while a gap of 3 min was given between concentrations of ligands.

### 2.5. Enzyme Assay for AChE Inhibition

AChE inhibitory activity of the extract and diosgenin was determined via the standard spectrophotometric method [18]. Certain modifications to the process were introduced, as described before [17]. Electric eel AChE (type VI-S) was utilized as an enzyme source, while ATCh iodide worked as the substrate. Ellman's reagent (DTNB) behaved as the chromogenic marker for determining enzyme inhibitory activity. For the working enzyme solution, sodium phosphate buffer (1 mM) was used. Enzyme concentrates were made and kept at -70°C; the substances under study were diluted on the test day.

The enzyme inhibitory tests were done in 96-well microtiter plates. Briefly, sodium phosphate buffer (140 *μ*l and 0.1 mM at pH 8.0), Tfg.Cr extract (20 *μ*l and diluted in 5% ethanol), and AChE (20 *μ*l) were dissolved and kept for 15 min at 25°C. Then, DTNB (10 *μ*l) was added. The interaction of chemicals initiated with the addition of 10 *μ*l of ATCh (0.71 mM). Breakdown of ATCh was quantified by determining synthesis of 5-thio-2-nitrobenzoate anion (yellow in color, formed due to interaction of DTNB and thiocholine) using SpectraMax microplate spectrophotometer (Molecular Devices, USA). The test for the substances under study was performed at least thrice. The preliminary difference was calculated as the difference in optical density/min and used in the following determination. The test reaction contained test samples, while the control lacked test substance. For comparison, the standard AChE inhibitor was physostigmine.

### 2.6. Molecular Docking and Dynamics Simulation

#### 2.6.1. Molecular Docking Protocol

The ligand diosgenin, a phytosteroid sapogenin (Figure 1), was considered with flexibility of a defined ligand-specific torsion tree. Torpedo californica acetylcholinesterase (TcAChE) structure retrieved from Protein data bank accession I.D. 7B2W was downloaded, protonated, and minimized prior to docking. Auto-Dock docking setup for TcAChE was asserted as rigid, and the grid spacing was applied onto the whole 3-dimensional protein structure to explore the putative binding sites. Auto-Dock algorithm having precalculated maps of each atom of the ligand with the pre-defined electrostatic potential was utilized. The autogrid algorithm implemented in Auto-Dock predicts the binding energy via the ligand conformation and contribution of each atom of a specified element with the specified grid point in the vicinity of the receptor. The grid box is generated around the active site of the TcAChE enzyme, allowing the docking software to look for all possible interactions between the ligands and the receptor. Other configurational settings were set as the default. Auto-Dock examined the intact 3D structure of AChE and ligand diosgenin in the prescribed grid spacing to search the space for docking. 2D and 3D molecular interactions of the protein-ligand complex was visualized to interpret the binding mode.

#### 2.6.2. Molecular Dynamics Simulation Setup

The investigated docked AChE-diosgenin complex was further expedited to spurt molecular dynamics (MD) production run via the Desmond simulation package. MD simulations of the AChE-diosgenin docked complex were accomplished to reveal the capability of the diosgenin inhibition in the acetylcholinesterase. Receptor topology was generated, and the SPC water model with the specified periodic boundary conditions at a distance of 1.0 nm was set to create an aqueous environment. Furthermore, the solvated receptor charges were neutralized by the addition of required Na^+^ or Cl^−^ ions. Subsequently, the default value of pressure and temperature was maintained as per the Parrinello-Rahman algorithm and Nose-Hoover temperature coupling method. The NPT ensemble was used for minimization and relaxation. The 100 ns production MD simulation run was executed with the interval record of 100 ps. Furthermore, simulated trajectories were analyzed and visualized via simulation interaction diagram (SID) module implemented in Desmond Schrödinger package to record the protein deviation, fluctuation, compactness, and hydrogen bond contacts with their occupancies, while ligand root means square deviation (RMSD), the radius of gyration (rGyr), torsional angle, molecular surface area (MolSA), solvent accessible surface area (SASA), and polar surface area (PSA) were also calculated during the production run of MD simulated time.

### 2.7. Result Representation

Data are shown as mean ± standard error of the mean (SEM; “*n*” is observations) and the effective concentration producing 50% inhibition (EC_50_) with 95% confidence intervals (CI). The graphs were constructed and analyzed utilizing the GraphPad program (GraphPad, USA). Statistical comparisons were made via two-way analysis of variance (ANOVA), and unpaired Student's *t*-test (*p* < 0.05) was taken as statistically different (GraphPad program).

## 3. Results and Discussion

This project was aimed at looking into some of the pharmacological activities of fenugreek seed extract. Fenugreek seeds are traditionally used in multiple GI conditions [1, 2, 4] while also regarded as useful as a CNS tonic to enhance memory [1, 5].

### 3.1. Effect of Tfg.Cr on Isolated Smooth Muscle Tissue Preparations

The 70% aqueous methanolic extract was investigated on the isolated rabbit and rat GI smooth muscle tissue preparations. The reason for selecting these tissue preparations was that rabbit jejunum, once isolated in a tissue bath under controlled conditions, elicits spontaneous contractions, and it is ideal for testing for potential GI spasmolytic agents [19, 20]. However, spasmogenic effects can also be investigated on rabbit jejunum. Tfg.Cr (0.1-10 mg/ml) did not exhibit a response when evaluated on spontaneous contractions of rabbit jejunum tissue preparations (*n* = 3; Figure 3(a)). There was neither a stimulant nor a relaxant effect seen with the extract (Figure 3(a)). This could be due to a number of reasons. The simplest reason could be that the plant extract just does not have any GI-active components which is why it did not exhibit any activity in this preparation. Other reasons are discussed below. In comparison to the extract, standard GI stimulants like ACh (10 *μ*M) and nicotine (10 *μ*M) both elicited an immediate stimulant effect on the resting spontaneous contractions (Figure 3(a)). The rat ileum, on the contrary, maintains a flat baseline and is ideal for screening spasmogenic activity [21]. When tried on this tissue, Tfg.Cr (0.1-10 mg/ml), similar to how it acted in rabbit jejunum, did not exhibit any effect (*n* = 3; Figure 3(b)). There was neither a stimulant nor a relaxant effect seen from Tfg.Cr (Figure 3(b)). On the contrary, standard gut spasmogenics like ACh (10 *μ*M) and histamine (10 *μ*M) both exhibited a sharp spasmogenic response on the baseline of rat ileum tissues (Figure 3(b)). These results are worth mentioning, keeping in mind that fenugreek seeds are used traditionally in constipation (spasmogenics help in constipation to increase the muscular tone) and diarrhoea (spasmolytics help to relax muscular tone in diarrhoea). The traditional use was an indication that the seed extract should have either spasmolytic or spasmogenic or even both activities, as we have shown so often from our studies [17]. The seeds are also known to contain choline, a quaternary ammonium compound that forms ACh in the body [22]. ACh is a major neurotransmitter in the biological systems as a modulator of GI motility and memory [22]. As to why we did not see any muscular tone modulatory effect of the extract in the isolated preparations, it could be that multiple chemical components in the seed extract balanced out a final effect due to their presence or maybe the GI-active component did not concentrate out in the 70% aqueous methanolic solvent that we used. There could even be a species-specific effect. We have shown, in the past, that plant extracts exhibit different effects in tissues from different species [21, 23, 24]. Whatever the reason may be, although negative, these findings help rule out several different variables for any future studies other researchers might decide to perform to further investigate the GI effects of this very popularly used herb.

### 3.2. Effect of Tfg.Cr and Diosgenin on Enzyme Assay for AChE Inhibition

**Figure 4:**
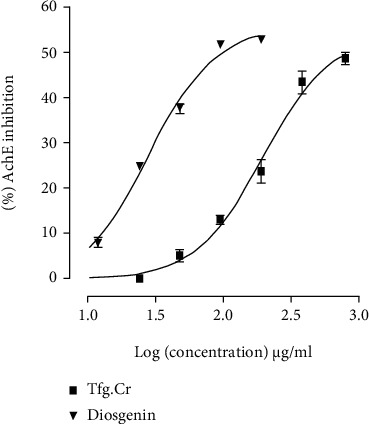
Curves showing the response of fenugreek crude extract (Tfg.Cr) and diosgenin on the in vitro enzyme assay for acetylcholinesterase (AChE) inhibition. Data presented are mean ± SEM, four observations; the two curves are statistically different (*p* < 0.001), two-way ANOVA.

Another reason for using the isolated tissue preparations was to see if the extract can show an ACh-like spasmogenic effect. This is because ACh is a GI stimulant and a major neurotransmitter implicated in the pathophysiology of Alzheimer's disease [25]. Chemicals with an ACh-like effect can help patients with memory disorders like Alzheimer's [22]. We have reported several ACh-like GI stimulant extracts with additional AChE inhibitory pharmacology [20, 26]. But as discussed, the extract did not exhibit any effect on the isolated tissues. Although when tested against the in vitro AChE enzyme assay, Tfg.Cr, in increasing concentrations (47-380 *μ*g/ml), inhibited the ACh-degrading enzyme AChE (Figure 4). The EC_50_ for this effect was 196.0 *μ*g/ml (152.6-251.9, *n* = 4). Tfg.Cr showed a maximum of 48.8 ± 1.3% (*n* = 4) inhibition of the enzyme (Figure 4). This inhibitory effect was like the effect exhibited by physostigmine, a standard AChE inhibitor, that showed its inhibitory effect with EC_50_ of 0.04 *μ*g/ml (0.04-0.04, 3 observations; data not shown). This action of fenugreek aligns with its folkloric consumption to enhance memory [1, 5]. AChE inhibitors are the mainstay of therapy for Alzheimer's disease, and currently, three out of the four clinically used Alzheimer's medications are AChE inhibitors [27]. One study in the literature [28] reports the AChE inhibitory effect of fenugreek but that study used an ethanolic standardized extract compared to our methanolic crude extract. This shows the widespread presence of AChE inhibitory constituents in fenugreek seeds. Recently, a couple of in vivo studies reported the positive effect of fenugreek on memory [29] and cognition [30].

To determine the responsible compound for this AChE inhibitory action, we tested diosgenin (Figure 1), a steroidal sapogenin reported in fenugreek [1, 2, 4]. Diosgenin, in increasing concentrations (11-190 *μ*g/ml), inhibited the AChE enzyme (Figure 4). This activity of diosgenin was significantly more potent than that of the extract (*p* < 0.001, two-way ANOVA). The EC_50_ of the suppressive action of diosgenin was 27.9 *μ*g/ml (24.9-31.1, *n* = 4). Diosgenin showed a maximum of 53.0 ± 0.4% (*n* = 4) inhibition of the AChE enzyme (Figure 4). The maximum inhibition of the AChE enzyme by diosgenin was significantly different from that of the crude extract (*p* < 0.01, unpaired *t*-test). We have earlier reported this AChE inhibitory effect of diosgenin [31], so in that sense, this finding regarding diosgenin is not novel. However, this shows that diosgenin is likely behind the AChE inhibitory effect observed with Tfg.Cr.

### 3.3. Molecular Docking Interpretation of Diosgenin Interactions with TcAChE

**Figure 5:**
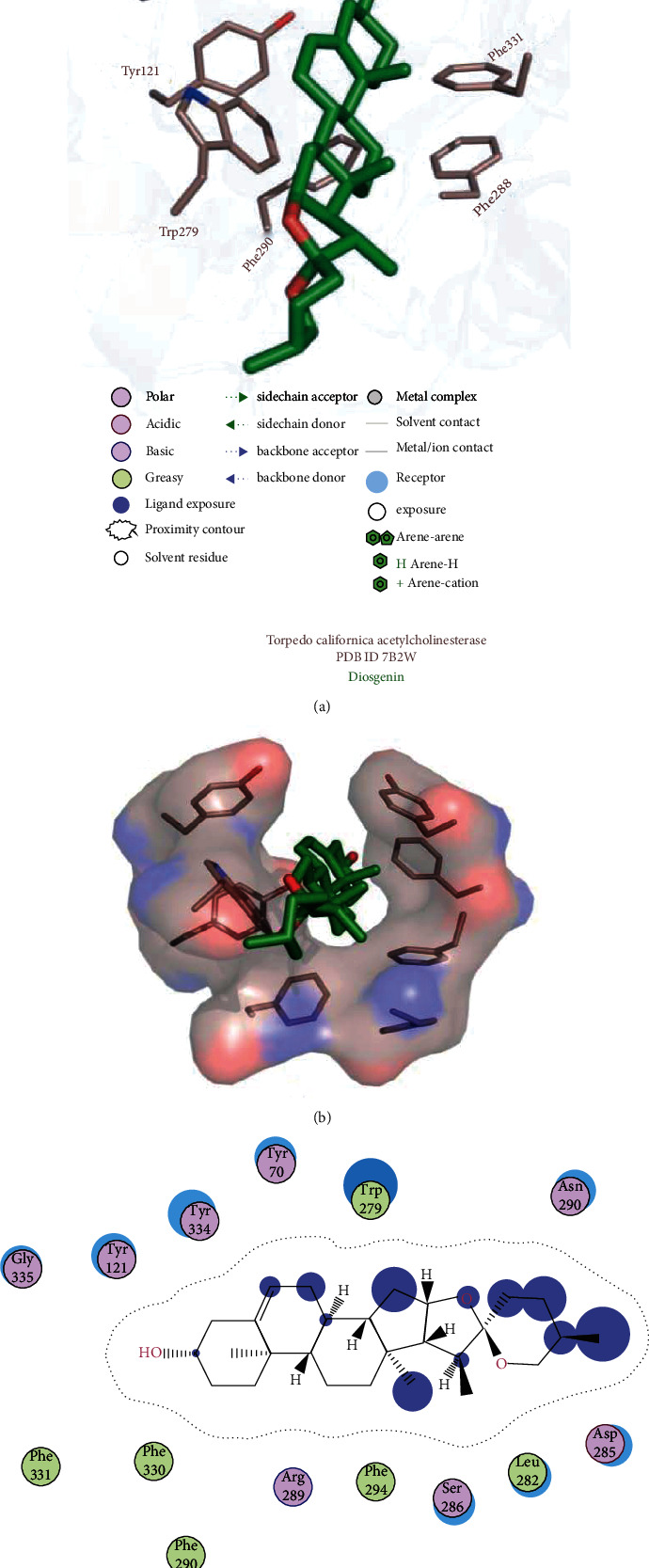
Postdocking analysis of Torpedo californica acetylcholinesterase with diosgenin. The green color represents ligand, while brown represents protein atoms.

A molecular modelling study was executed to interpret the binding mode analysis of diosgenin into the possible binding cavity of the TcAChE receptor 7B2W. The docking output with putative interaction site with the highest *Δ*G binding energy threshold of −10.7 kcal/mol was observed among all the ranked poses based on the estimated interaction energy. The top ranked conformation of diosgenin according to the highest *Δ*G binding energy was elected for molecular investigation. Auto-Dock Vina top most ranked pose output revealed that diosgenin established hydrogen-mediated, hydrophobic, and pi-pi stacking contacts with the aromatic amino acid residues of TcAChE of the peripheral anionic site (Tyr70, Tyr121, Trp279, and Tyr334), an anionic subsite (Phe330), catalytic site (Phe331), and acyl pocket residues (Phe288 and Phe290) as illustrated in Figure 5. PAS anionic site and anionic subsite residues have been previously reported for the CNS activity. These amino acids Tyr70, Tyr121, Trp279, Phe288, Phe290, Phe331, and Tyr334 which directly involve in mediating hydrophobic interactions were discovered in case of diosgenin which were reported for CNS activity in literature.

### 3.4. MD Analysis

Molecular dynamic simulation data analysis is the basic quantitative evaluation parameter to analyze the stability, instability, deviations, fluctuations, compactness, and molecular interaction contact analysis of the protein and ligand via conformational geometries and dynamic vibrations and motions.

#### 3.4.1. AChE-Diosgenin Complex Deviation and Fluctuation

**Figure 6:**
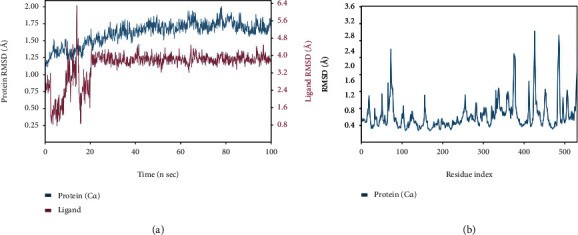
Deviation and fluctuation analysis in the TcAChE-diosgenin complex. (a) RMSF plot of the protein-ligand complex. Blue color indicates the alpha-carbon backbone of the protein, while maroon color indicates RMSD upon ligand fitting. (b) RMSD of the backbone carbon-alpha atoms of protein and heavy atom ligand.

RMSD was used to record the dislocation of the atoms present within the protein and ligand during MD simulated time with the initially generated frame of the pre-MD production run. RMSD plot analyzed for the protein backbone carbon-alpha atoms and the heavy atoms of the ligand during 100 ns of MD executed production runs. RMSD plot of TcAChE receptor 7B2W-diosgenin complex as depicted in Figure 6(a) demonstrated that AChE receptor deviations were noted within the range of 1.2 Å to 1.75 Å during the 100 ns of the trajectories record. In contrast, more deviations were observed between 65 ns and 75 ns with the deviation of ±2 Å. In the case of ligand fitting, RMSD with the heavy atoms of diosgenin in the 7B2W receptor showed more distortions in the initial 5 to 25 ns of the production run within the range of 1.6 Å to 6.4 Å. In comparison, the stability was almost attained after 25 ns to 100 ns near to ±4 Å. A slight or minor deviation was noted at 65 ns as the same noted in the protein RMSD. Lig fit Prot plot revealed that ligand had been slightly diffused away from its binding pocket. Root mean square fluctuation of the protein-ligand complex of 537 amino acid residues was also analyzed during 100 ns of MD run. The influence of residue fluctuations was also noted for this 7B2W-diosgenin complex with Tyr70, Trp84, Trp279, and Phe288-Tyr334. Significant fluctuation at Tyr70 amino acid residue within the PAS region was observed. Minor fluctuations were observed with the other interacting PAS and an anionic subsite residue. The low deviation via RMSD and less fluctuation via RMSF results suggested that TcAChE, when complexed with the diosgenin, showed stability with the respective protein and its binding cavity.

#### 3.4.2. Protein-Ligand Interaction Contact Analyses

**Figure 7:**
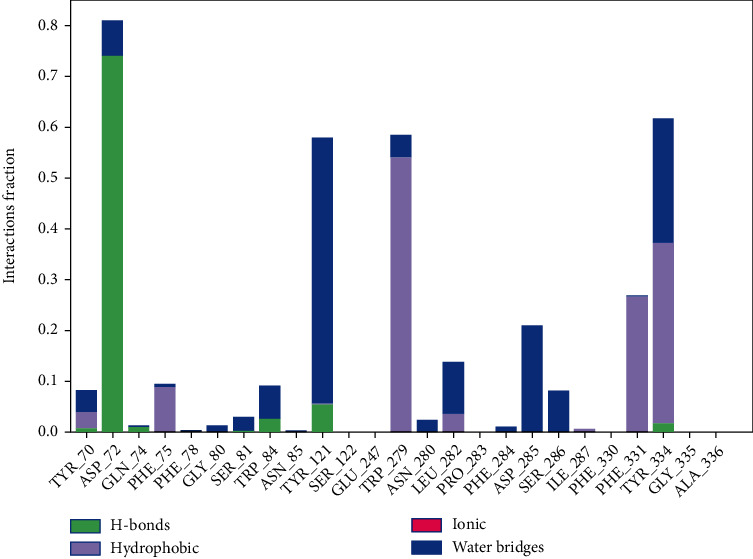
Histogram analysis of the interacted fraction pattern of amino acid residues with the ligand.

**Figure 8:**
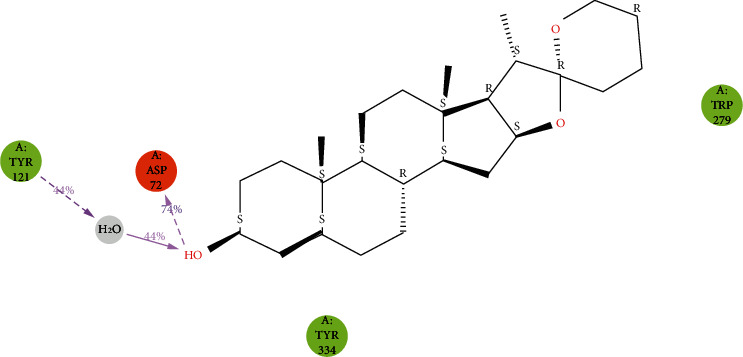
2-dimensional interaction pattern observed during 100 ns of MD production run.

The stability of the TcAChE-diosgenin was estimated via a protein-ligand contact histogram plot to check the overall protein-ligand contacts with the active-site residues in terms of interaction fraction pattern. These protein-ligand contacts were also inspected on the simulated trajectories of 100 ns of the production run. The interaction patterns follow H-bonds, hydrophobic, ionic, and water bridging-mediated interactions. H-bonding interactions play a crucial role in drug designing, but in this contrast, hydrogen bonding occurred as 0.74% with Asp72 and <0.1% with the Tyr121 and Tyr334. Hydrophobic contacts were established with Tyr70, Phe75, Trp279, Phe331, and Tyr334 while 0.55% hydrophobic contact with Trp279 and 0.3% and 0.35% fraction contact with Phe331 and Tyr334 were noted. Trp84, Tyr121, and Tyr334 possessed weak hydrogen bonding interactions. Asp72 showed maximum H-bond occupancy throughout the simulation, but in the last 25 ns, MD run. Its contact with diosgenin was diminished and converge into the water-mediated bridging interactions. At the same time, other residues which are mediating interactions with negligible hydrogen bond occupancies were observed negligible as implemented in Figure 7. 2D protein-ligand interaction pattern was also checked for further explanation as depicted in Figure 8. It is also evident by the 2D protein-ligand contact diagram, which was also showing that hydroxyl moiety of the diosgenin-mediated H-bond with Asp72 persists to 74% during simulation while the water-bridge interaction with the Tyr121 persisted 44%.

**Figure 9:**
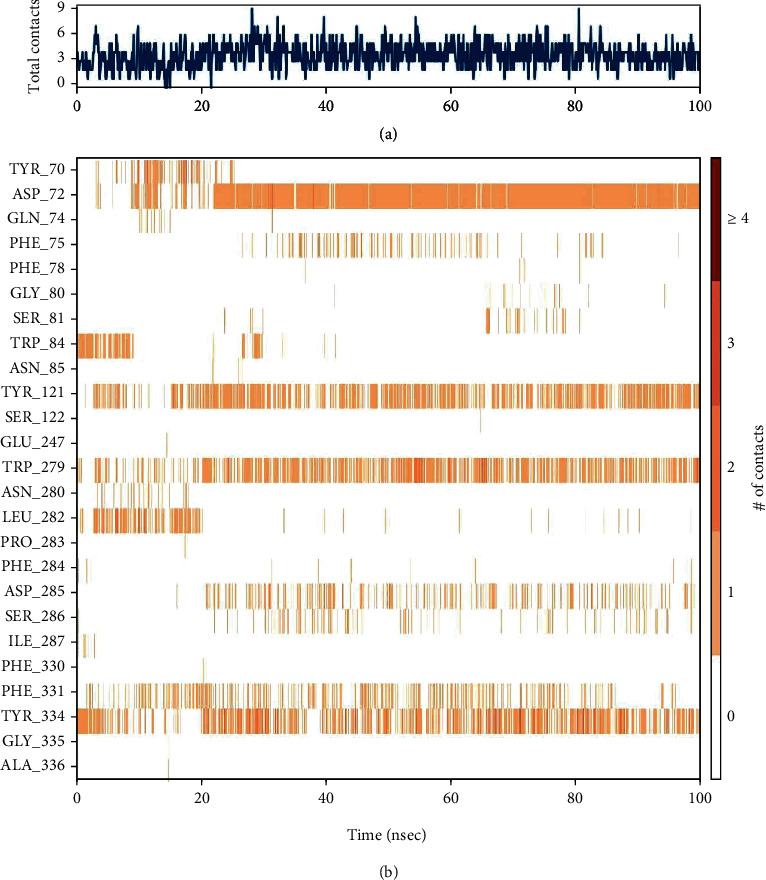
(a) Timeline representation of the total number of molecular interaction contacts in each trajectory frame. (b) The number of interactions with the active-site residues in each frame of the simulated 100 trajectory frames.

A timeline representation of the above-mentioned receptor-ligand histogram was further analyzed to check the total number of interaction or molecular contacts in each trajectory record frame of all amino acid residues of the TcAChE, further clarified by the timeline exhibit of active-site residues of the TcAChE-diosgenin complex. The top panel of the timeline representation, as depicted in Figure 9(a), demonstrated the total number of exhibited molecular contacts in each trajectory record frame. Throughout the 100 ns of the production run, almost 05 intermolecular contacts persisted while the increase in molecular contacts was noted at 30 and 80 ns. During this phase of the MD run, it showed variation in establishing any molecular interactions that raised from 5 to 9. Furthermore, we noted which amino acid residues mediated intermolecular contacts in each trajectory throughout the simulation, as mentioned in Figure 9(b). Asp72, Tyr121, Trp279, Phe331, and Phe334, the peripheral active-site gorge residues, interacted throughout the simulation, while Tyr70, Phe75, Gly80, Ser81, Trp84, Leu282, Asp285, Ser286 interacted very few or showed inconsistent molecular interaction as indicated by light orange color, which means a single contact obtained during simulation. Subsequently, Asp72 showed a dark band of orange color, showing that it interacted in high occupancy with more molecular contacts throughout the simulation, which validates that the crucial active-site amino acid residues have more interactions with diosgenin. It verified that approximately all probable positional geometries were achieved during this highly occupied H-bonds as described in the aforementioned histogram plot. Despite Asp72, Tyr121, Trp279, Phe331, and Phe334 showed variation sometimes single or more contacts throughout the simulation, these molecular contacts showed inconsistency during 100 ns of MD production run each frame trajectory record.

#### 3.4.3. Detailed Analyses of the Diosgenin Properties throughout the Simulation

**Figure 10:**
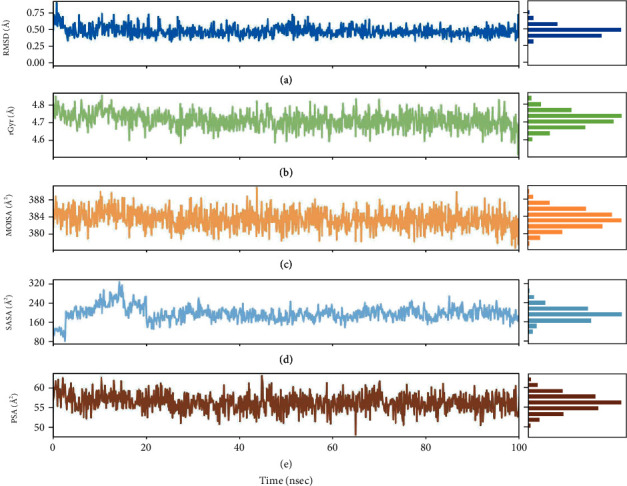
The ligand property analyses of 7B2W-diosgenin complex throughout the trajectory of 100 ns simulation run. (a) Ligand root means square deviation (RMSD), (b) radius of gyration (rGyr), (c) molecular surface area (MolSA), (d) solvent accessible surface area (SASA), and (e) polar surface area (PSA).

**Figure 11:**
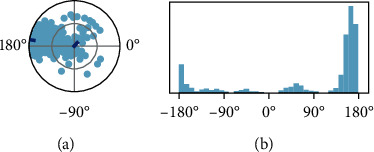
Ligand torsional profile of the rotatable bonds present in diosgenin during simulation. (a) The conformation of torsion during simulation. (b) The bar plot demonstrating the probable density of the torsions present in the ligand.

The ligand properties were studied carefully to understand the conformational repositions utilizing the reference of pre-MD production data. For this purpose, all atoms of the diosgenin used for the detailed analysis such as RMSD, compactness or extendedness, MolSA determination via 1.4 Å probe radius, SASA, the PSA were estimated as shown in Figure 10, and the torsional angle profile of the rotatable bonds present in the diosgenin was also critically analyzed as explained in Figure 11.

The RMSD of the ligand showed stability throughout the simulation, while minor fluctuations were seen at 50-55 ns after reaching the equilibrium position after 5 ns. The ligand RMSD lay around 0.25 Å to 0.8 Å, while RMSD attained around 0.6 Å during the whole simulation shown in Figure 10(a). The rGyr was also measured by which the compactness of the ligand was analyzed. The rGyr of the diosgenin slightly fluctuated to 40 ns simulation, later slowly attained equilibrium. The ligand showed an rGyr range of about 4.6 Å to 4.8 Å, and the equilibria attained around ±4.7 Å (Figure 10(b)). Another property MolSA was also determined with a 1.4 Å probe radius. MolSA of ligand seemed stable during simulation, although there was a little less variation in 45 ns and 85 ns trajectory records. The range of MolSA is around 380Å^2^ to 388Å^2^, and the equilibrium achieved around 385Å^2^ is visualized in Figure 10(c). The SASA by H_2_O solvent was also noted. The SASA denoted that the raised value from 80 to 320 Å^2^ until 20 ns simulation and then afterward became persistent until the end of the simulation with the range of 200Å^2^ (Figure 10(d)). The PSA was also estimated for diosgenin molecules influenced by the polar O_2_ and N atoms. The PSA was in the range of 50Å^2^-60Å^2^ throughout the simulation, but there were visible fluctuations occurred at 46 ns to 65 ns simulation period, while the equilibrium attained around 56Å^2^ inspected in Figure 10(e). The above-mentioned studied ligand properties show that there were some fluctuations in the initial or intermediate recorded trajectory frames, which gradually achieved equilibrium till the completion of the simulation, which demonstrated the stability of diosgenin in the active site of the gorge region TcAChE.

Dial plot illustrated that the torsion angle variations persisted throughout the whole MD production runs. The bar plot emphasized the dial plots by indicating the probable density of the torsions via utilizing each rotatable bond present in the ligand, as depicted in Figure 11. It showed that the torsional angle of the rotatable bond lay between 160 to 180 degrees of angular rotation, while the occupancy showed at 170 degrees of angular rotation. Histogram and torsion interactions may reflect conformational strain that ligand faces to sustain a protein-bound conformation.

### 3.5. Pre- and Post-MD Binding Mode Analysis of TcAChE-Diosgenin Complex

Molecular docking and post-MD results revealed that diosgenin penetrated in the active-site gorge region of TcAChE and showed almost similar molecular interactions in the static and dynamic mode. The peripheral anionic site aromatic residues Trp279 and Tyr334 showed hydrophobic or pi-pi stacking contacts with diosgenin in both pre- and post-MD stages. Subsequently, new molecular interactions were also observed and mediated hydrogen bond during 100 ns MD run with the hydroxyl moiety of diosgenin via Asp72 with the distances of 1.68 Å and 2.84 Å and Ser81 with the distances of 2.93 Å. The OH group interacted with Asp72- and Ser81-mediated H-bonding interactions could be the reason for enhancing the binding affinity of diosgenin in TcAChE.

## 4. Conclusion

These results show that although fenugreek is so popularly used traditionally in GI disorders, it exhibited neither a stimulant nor a relaxant effect on the GI smooth muscles. This is an important finding as it rules out several variables and gives valid information to other researchers to try either a different solvent system for the extraction or different animal species to investigate any potential GI effects of this herb. Isolated smooth muscle preparations not only help to elucidate GI effects but also help look for ACh-like effects, which are also relevant in Alzheimer's disease pharmacology. Although it did not affect the isolated tissues, the extract did exhibit an inhibitory effect on the AChE enzyme assay, providing a rationale for its traditional consumption in memory loss. Diosgenin, a known chemical component of fenugreek, also showed a much more potent inhibitory effect on the AChE enzyme assay, indicating that it must be the responsible compound in fenugreek for this effect. After applying in silico approaches, we concluded that diosgenin permeated the active-site gorge of TcAChE and displayed substantially identical molecular interactions in the static and dynamic states obtained from molecular docking and post-MD findings.

## Data Availability

All data is provided in the study.
